# Methods to Evaluate COVID-19 Preventive Hygiene Programs: Observed Mask Wearing, Handwashing, and Physical Distancing Behaviors in Public Indoor Spaces in Democratic Republic of the Congo

**DOI:** 10.4269/ajtmh.22-0214

**Published:** 2022-10-03

**Authors:** Christine Marie George, Kelly Endres, Presence Sanvura, Camille Williams, Raissa Boroto, Claude Lunyelunye, Jean Claude Bisimwa, Jessy Timsifu, Brigitte Munyerenkana, Justin Bengehya, Ghislain Maheshe, Alain Mwishingo, Cirhuza Cikomola, Lucien Bisimwa

**Affiliations:** ^1^ Department of International Health, Johns Hopkins Bloomberg School of Public Health, Baltimore, Maryland;; ^2^ Center for Tropical Diseases & Global Health, Université Catholique de Bukavu, Bukavu, Democratic Republic of the Congo;; ^3^ Bureau de l’Information Sanitaire, Surveillance Epidémiologique et Recherche Scientifique, Division Provincial de la Santé Sud Kivu, Ministère de la Santé, Bukavu, Democratic Republic of the Congo;; ^4^ Faculty of Medicine, Université Catholique de Bukavu, Bukavu, Democratic Republic of the Congo

## Abstract

The objective of our study was to develop and test observational methods to evaluate COVID-19 preventive hygiene behaviors and physical distancing, and to evaluate the effectiveness of a government mandate on indoor fully covered mask wearing. An observational study was conducted of 4,736 individuals from April to October 2021 using 5-hour and rapid (10-minute) structured observations and spot checks to evaluate mask-wearing, handwashing, and physical-distancing behaviors, and the functionality of handwashing stations in 161 indoor public spaces across Bukavu, Democratic Republic of the Congo (DRC). Sixteen percent of individuals entering indoor public spaces were wearing a mask that fully covered their nose and mouth (fully covered mask wearing). Fully covered mask wearing was lowest inside schools (1%), universities (2%), religious establishments (22%), and health facility wards (28%). Overall physical distancing of more than 1-m inside indoor public spaces was 22%, and was lowest inside schools and religious establishments (7%). Thirty-nine percent of handwashing stations had water and a cleansing agent present. Ten percent of individuals washed their hands with a cleansing agent before entering an indoor space. Overall, fully covered mask wearing was similar for 5-hour and rapid structured observations (16% versus 15%). The odds of fully covered mask wearing was significantly greater with increased government enforcement of mask wearing in public spaces through fines (odds ratio, 2.72; 95% CI, 1.02–7.30). This study presents rigorous methods using structured observations to assess government mandates and programs on COVID-19 preventive hygiene behaviors in indoor public spaces in settings globally.

## INTRODUCTION

As of May 13, 2022, the Democratic Republic of the Congo (DRC) has reported 87,023 cases and 1,337 deaths of SARS-CoV-2, the virus that causes COVID.^1^ This, however, is likely an underestimate, with surveys in the DRC finding a SARS-CoV-2 seroprevalence of 17%.^2^ COVID-19 vaccine availability is currently limited in the DRC, with 1.2% of the population vaccinated.^1^ Mask wearing covering the nose and mouth, physical distancing, and handwashing with a cleansing agent in indoor public spaces are critical to prevent the spread of COVID-19.^3^^–^^5^ The WHO has issued guidelines on hygiene behaviors to prevent the spread of COVID-19.^6^ For physical distancing, the WHO advises maintaining a greater than 1-m physical distance from others, including outside and when masks are worn.^4^ Correct mask wearing is defined as wearing a mask that covers the nose and mouth.^3^ Mask wearing is recommended in indoors public spaces and outdoors when greater than 1-m physical distancing cannot be maintained.^3^ In addition, the WHO recommends regularly cleaning hands with an alcohol-based hand rub or soap and water at key times, including before and after touching common surfaces.^5^

Rigorous methods are needed to assess COVID-19 preventive hygiene behaviors to determine compliance with government mandates related to COVID-19 prevention and for COVID-19 preventive hygiene programs delivered at the national and sub-national level to be evaluated. Globally, very few studies have used direct observation to assess COVID-19 preventive behaviors.^7^^–^^23^ Most studies on the rates of COVID-19 preventive hygiene behaviors focus exclusively on self-reported behaviors, which is prone to reporting bias. Of the observational studies available, most focused on mask wearing, with only a handful of studies conducted in low- and middle-income countries,^8^^,^^11^^,^^13^^,^^15^^–^^17^^,^^23^ and only half of studies were conducted in indoor public spaces.^7^^–^^10^^,^^12^^,^^14^^,^^15^^,^^17^^,^^23^ Furthermore, no published study has conducted structured observation to assess mask-wearing, handwashing, and physical-distancing behaviors together in indoor public spaces—a high-transmission environment for COVID-19.

Public health authorities in the DRC have issued government mandates and recommendations on physical distancing (> 1-m) and handwashing with a cleansing agent when entering indoor public spaces, as well mask mandates in August 2021 with fines for noncompliance of 5,000 Congolese francs (US$2.50).^24^^,^^25^ The objective of our study was to develop and test observational methods to evaluate COVID-19 preventive hygiene behaviors and physical distancing, and evaluate the effectiveness of a government mandate on indoor fully covered mask wearing. We conducted an observational study of 4,736 individuals using structured observation and spot checks to evaluate mask-wearing, handwashing, and physical-distancing behaviors, and functionality of handwashing stations in 161 indoor public spaces across Bukavu, DRC. In this study, we also compared the level of agreement between 5-hour structured observations and 10-minute rapid structured observations for these behavioral outcomes inside indoor public spaces.

## METHODS

### Study design.

This observational study of 4,736 individuals in 161 unique indoor public spaces was conducted in the city of Bukavu, South Kivu Province, DRC. The sample size was based on the number of individuals observed during structured observations. The number of locations included in the study, and the number included in structured observations, was based on the maximum sample size our project budget could accommodate and ensure coverage across Bukavu. Fifteen indoor public location types were included: shops, banks, offices, wards and main entrances of health facilities, beauty salons, supermarkets, schools, restaurants, large retail centers, universities, religious establishments (e.g., churches and a mosque), saunas, physical therapy offices, and gyms. Activities at religious establishments were conducted throughout the week from 12:00 noon to 5:00 pm; for the mosque in Bukavu, activities were conducted on a Friday. Spot checks were conducted at 10 or more of each of the 15 indoor public location types (e.g., shops, banks); 161 unique locations had spot checks conducted. No locations were visited more than once for spot checks and structured observations. Structured observations were conducted in a randomly selected subset of five or more of each of the 15 indoor public location types. Locations were selected if a handwashing station was present at an indoor entry. The list of indoor public space locations was compiled based on a scouting exercise using convenience sampling by the study team, during which a research officer went across the city and made a list of locations where handwashing stations were present. Our goal was at least five 5-hour and rapid structured observations at each type of location. Random selection for structured observations was performed based on the list of all public locations with handwashing stations at entrances. Research assistants received 1 week of training on how to conduct structured observations and spot checks.

### Procedures.

Structured observations and spot checks were conducted from April to October 2021 by our team of five research staff. Spot checks of handwashing stations (∼10 minutes in length) were conducted at the entrance of indoor public spaces before structured observations. Handwashing stations were checked for water and cleansing agents (bar soap, liquid soap, soapy water, and hand sanitizer), functionality (whether stations had a functional tap and water present), and handwashing with a cleansing agent or COVID-19/mask-wearing signage. The chlorine concentrations of handwashing stations were assessed using a digital colorimeter (Hach, Loveland, CO). The U.S. Centers for Disease Control and Prevention^26^ recommends a chlorine concentration for hand cleansing of 0.05% or 500 mg/L.

Five-hour structured observation was conducted by trained research assistants at the entrances of each indoor public space to observe mask-wearing, handwashing with a cleansing agent, and physical-distancing behaviors upon entry of indoor spaces. Staff sat in an outdoor inconspicuous location away from the entrance of public spaces to avoid attracting attention. A structured form was used to collect information on the following WHO-recommended COVID-19 preventive hygiene behaviors^3^^–^^5^ upon entry of public indoor spaces: 1) type of mask-wearing behavior (mask fully covered defined as nose and mouth fully covered), 2) handwashing with a cleansing agent (bar soap, liquid soap, powdered soap, soapy water from a bottle, soapy water in any container, ash, mud/soap, hand sanitizer, and chlorinated water); and 3) physical distancing (defined as > 1-m of space between individuals). Chlorinated water during structured observation was assessed by a sign being present on the handwashing station indicating that chlorinated water was present. In addition to 5-hour structured observations, rapid structured observations were also conducted in indoor public spaces, during which research assistants entered indoor public spaces and assessed the mask-wearing and physical-distancing behaviors of all those inside. These rapid structured observations were approximately 10 minutes in length to limit time research staff spent in indoor public spaces. Multiple research staff observed individuals simultaneously in larger indoor public spaces such as churches to limit the time staff needed to spend indoors. These observations were done on a separate day from when the 5-hour structured observations were conducted.

### Statistical analysis.

Descriptive statistics were calculated on physical-distancing (> 1-m), fully covered mask-wearing, and handwashing with a cleansing agent; as well as the percentage of locations with a handwashing station with a cleansing agent and water present, and handwashing with a cleansing agent and COVID-19 signs. To assess whether COVID-19 preventive hygiene behaviors varied over time, logistic regression models were performed using generalized estimating equations to adjust for clustering at each location with fully covered mask wearing, physical-distancing, and handwashing with a cleansing agent as the binary outcome, and study month as the predictor for 5-hour structured observations. To determine whether there were differences in COVID-19 preventive hygiene behaviors between locations with and without a COVID-19/mask-wearing sign being present, logistic regression models were used with generalized estimating equations to adjust for clustering at each location with fully covered mask wearing, physical-distancing, and handwashing with a cleansing agent as the binary outcome, and a COVID-19/mask-wearing sign being present as the predictor for 5-hour structured observations.

### Ethics approval.

Study procedures were approved by the research ethical review committees of the Catholic University of Bukavu, University of Kinshasa, and the Johns Hopkins Bloomberg School of Public Health. Because no identifying information was collected from individuals in public spaces, no informed consent was required.

## RESULTS

A total of 4,736 individuals in indoor public spaces were observed during the structured observations conducted in Bukavu, South Kivu Province, DRC. There were 161 unique locations with spot checks. Of these locations, 89 unique locations had 5-hour structured observations, and a subset of 75 of these 89 locations had rapid structured observations. A total of 3,781 individuals were observed during 5-hour structured observations; 955 individuals were observed during the rapid structured observations. Female participants represented 41% (1,568 of 3,781) and 48% (458 of 955) of individuals in the 5-hour and rapid structured observations respectively.

### Observed mask wearing.

For the 5-hour structured observations, 16% (620 of 3,781) of individuals entering indoor public spaces were wearing a mask that fully covered their nose and mouth (Table 1). Twelve percent of individuals (467 of 3,781) observed exhibited partial mask wearing (only mouth or nose covered, mask around neck, or mask around chin). Fully covered mask wearing was greatest when entering physical therapy offices (50%), followed by gyms (42%), and religious establishments (34%). The lowest percentage of fully covered mask wearing when entering indoor public spaces was found at beauty salons (5%), and schools and restaurants (6%). Fully covered mask wearing when entering health facilities and universities was 13%.

**Table 1:** Percentage fully covered mask wearing (mask fully covering nose and mouth), by location type, for entering indoor spaces (5-hour structured observation) and inside indoor spaces (rapid structured observations) *N* = 4,736

Location type	5-Hour structured observation of % fully covered mask wearing* (*n* = 3,781)		Rapid structured observation of % fully covered mask wearing*† (*n* = 955)		Difference from entering to inside (%)‡
	Total unique locations	Entering indoor space (%)	Total unique locations	Inside indoor space (%)	
Bank	6	31	5	48	17
Beauty salon	5	5	5	3	–2
Gym	5	42	5	0	–42
Health facility, main entrance	9	13	5	12	–1
Health facility, ward entrance	5	22	5	28	6
Large retail center	8	11	5	4	–7
Office	5	11	5	8	–3
Physical therapy office	5	50	5	13	–37
Religious establishment	8	34	5	22	–12
Restaurant	5	6	5	0	–6
Sauna	5	21	5	0	–21
School	7	6	5	1	–5
Shop	5	7	5	32	25
Supermarket	5	11	5	4	–7
University	6	13	5	2	–11
Overall	89	16	75	15	–1

* Defined as wearing a mask that covers the mouth, nose, and chin, according to the WHO definition for correct mask wearing.

† Rapid structured observations were conducted at the same location, but on a different day after the 5-hour structured observation.

‡ The percentage of individuals observed at each location wearing mask correctly.

For the rapid structured observations, overall, 15% of individuals (148 of 955) exhibited fully covered mask wearing and 12% exhibited partial mask wearing when inside indoor public spaces. Fully covered mask wearing was greatest inside banks (48%) and lowest at restaurants, saunas, and gyms (0%). Fully covered mask wearing was 1% inside schools, 2% at universities, 12% in indoor entrances of health facilities, 28% in health facility wards, and 22% in religious establishments. The largest increases in mask wearing from entering an indoor space (5-hour structured observations) to being inside an indoor space (rapid structured observations) was for shops, with an increase from 7% to 32%. The largest decreases in mask wearing were for gyms, with a decrease from 42% to 0%, and for physical therapy offices, with a decrease from 50% to 3%.

### Physical-distancing.

For 5-hour structured observations, 42% of individuals (1,585 of 3,781) maintained a physical distance of more than 1-m when entering indoor public spaces during (Table 2). This was greatest for physical therapy offices (59%) and supermarkets and saunas (57%). Physical-distancing when entering indoor spaces was lowest for restaurants (22%), universities (31%), and religious establishments (34%).

**Table 2:** Physical distancing of greater than 1-m by location type for entering indoor spaces (5-hour structured observation) and indoors (rapid structured observations; *N* = 4,736)

Location type	5-Hour structured observation of physical distance of > 1 m* (*n* = 3,781)		Rapid structured observation of physical distance of > 1 m* (*n* = 955)		Difference from entering to inside (%)†
	Total locations	Entering indoor space (%)	Total locations	Inside indoor space (%)	
Bank	6	52	5	41	–11
Beauty salon	5	35	5	13	–22
Gym	5	56	5	95	39
Health facility, main entrance	9	38	5	47	9
Health facility, ward entrance	5	46	5	47	1
Large retail center	8	45	5	8	–37
Office	5	38	5	35	–3
Physical therapy office	5	59	5	63	4
Religious establishment	8	34	5	7	–27
Restaurant	5	22	5	21	–1
Sauna	5	57	5	55	–2
School	7	43	5	7	–36
Shop	5	38	5	36	–2
Supermarket	5	57	5	40	–17
University	6	31	5	27	–4
Overall	89	42	75	22	–20

* Individuals were considered physically distanced if more than 1 m from other people, according to the WHO definition for physical distancing.

† The percentage of individuals observed at each location who were physically distanced.

For rapid structured observations, overall, 22% of individuals (203 of 955) maintained the 1-m physical distance in indoor public spaces. Physical-distancing was lowest in schools and religious establishments (7%), and highest for gyms (95%). For health facilities, physical-distancing was 28% inside wards, and 47% inside the main entrances. Universities had 27% of individuals maintaining physical-distancing. The greatest increase in physical-distancing from entering indoor spaces (5-hour structured observations) to being inside indoor public spaces (rapid structured observations) was for gyms: from 56% to 95%. The largest decreases were for schools, from 43% to 7%; for religious establishments, from 34% to 7%; and for large retail centers, from 45% to 8%.

### Handwashing with a cleansing agent.

For 5-hour structured observations, 10% of individuals (386 of 3,781) washed their hands with a cleansing agent before entering an indoor space. This percentage was greatest in banks (41%), physical therapy offices (28%), and religious establishments (21%). The percentage was lowest at shops, with only one individual washing their hands with a cleansing agent before entering; at beauty salons (2%); and at offices, supermarkets, schools, saunas, and health facility ward entrances (3%). Of the 386 handwashing-with-a-cleansing-agent events, the most commonly used cleansing agent was liquid soap (46%), followed by chlorinated water (indicated by a sign on the handwashing station, 40%), bar soap (10%), hand sanitizer (8%), and soapy water (water and detergent powder) and detergent powder (< 1%).

### Spot checks of handwashing stations.

**Figure 1.**
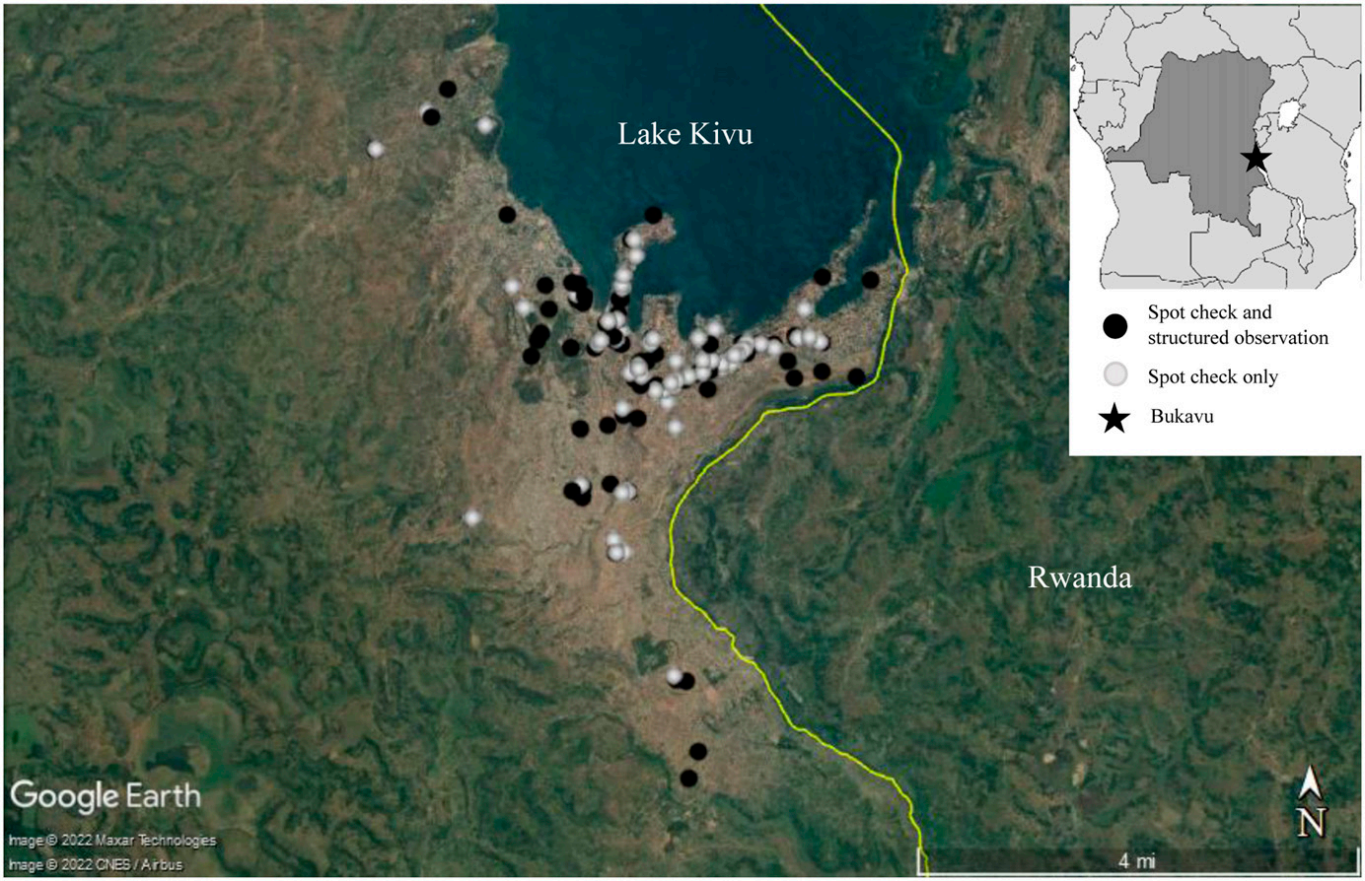
Map of study site of 161 locations where spot checks and structured observations were conducted. Dots represent locations where activities occurred. Bukavu is located in eastern Democratic Republic of the Congo, south of Lake Kivu, on the border with Rwanda. This figure appears in color at www.ajtmh.org.

A total of 161 locations were included in spot checks (Figure 1). These locations had 203 handwashing stations at indoor entrances; 29 locations had multiple handwashing stations. Eleven locations that originally had handwashing stations during initial scouting did not have these present when spot checks were conducted. Forty percent of handwashing stations (81 of 203) had water and a cleansing agent present. This percentage was greatest at restaurants (82%), followed by banks and offices (55%), and religious establishments (50%). The location types with the lowest number of handwashing stations with water and a cleaning agent present were universities (18%), beauty salons (22%), physical therapy offices (23%), schools (24%), and the entrance of health facility wards (32%). Fifty-two percent of handwashing stations (106 of 203) had water only, 2% (4 of 203) had a cleansing agent present but no water, and 6% (12 of 203) had neither water nor a cleansing agent. The most common cleansing agent was liquid soap (25%), followed by bar soap (14%), soapy water (water and detergent powder; 2%), hand sanitizer (1%), and chlorinated water (< 1%). Eighty-three percent of handwashing stations (169 of 203) were considered functional, which is defined as having a tap without leaks and the presence of water. Seventeen percent of locations (28 of 161) had a COVID or mask-wearing sign present at a handwashing station. This percentage was the greatest at health facility ward entrances (42%) and health facility main entrances (38%) (Supplemental Table 1). Eighteen percent of locations (29 of 161) had a handwashing station with a cleansing agent sign present at the handwashing station. This percentage was greatest at health facility ward entrances (50%) and health facility main entrances (38%) (Supplemental Table 1). No handwashing stations had free available chlorine concentrations greater than or equal to 0.05%. The average free available chlorine concentration detected was 0.18 mg/L (range, 0–8.6 mg/L). Nine percent of handwashing stations (19 of 203) had broken taps, 8% (17 of 203) had items other than water present in the basin (e.g., clothing, trash, supplies), and 11% (22 of 203) did not have a basin underneath to collect water.

### Regression analyses.

During August 2021, when the DRC government implemented a fine for noncompliance with the mask-wearing mandate in public spaces, the odds of fully covered mask wearing were significantly greater than when the study started in April 2021 (odds ratio, 2.72; 95% CI, 1.02–7.30) (Table 3). No other significant differences were observed between COVID-19 preventive hygiene behaviors and study month. No significant differences were observed between the presence of COVID/mask-wearing signage and any of the COVID-19 preventive hygiene behaviors.

**Table 3:** Trends in COVID-19 preventive hygiene behaviors over time when entering indoor spaces during 5-hour structured observation

Study month, 2021	Fully covered mask wearing*				Washing both hands with a cleansing agent†				Physical distancing > 1 m‡			
	%	*n*	*N*	Odds ratio (95% CI)	%	*n*	*N*	Odds ratio (95% CI)	%	*n*	*N*	Odds ratio (95% CI)
April	18	54	308	Reference	8	25	300	Reference	35	107	308	Reference
May	5	19	384	0.29 (0.07–1.27)	4	15	372	0.55 (0.09–3.49)	38	145	384	1.78 (0.55–5.81)
June	10	202	1,973	0.51 (0.20–1.31)	9	165	1,887	1.16 (0.39–3.44)	45	893	1,973	2.16 (0.77–6.01)
July	23	141	604	1.31 (0.45–3.76)	10	57	572	1.12 (0.35–3.65)	40	242	604	1.77 (0.61–5.14)
August	41	187	458	**2.72 (1.02**–**7.30)**	26	116	448	1.78 (0.46–6.93)	36	165	458	1.93 (0.64–5.81)
September	31	17	54	1.46 (0.27–7.92)	15	8	54	1.20 (0.17–8.50)	61	33	54	4.53 (0.85–24.07)

Bold text indicates significance at *P* < 0.05.

* Defined as wearing a mask that covers the mouth, nose, and chin, according to the WHO definition for correct mask wearing.

† Cleansing agents were bar soap, liquid soap, soapy water, and hand sanitizer. Percentages indicate the percent of individuals observed at each 5-hour structured observation.

‡ Individuals were considered physically distanced if more than 1-m from other people, according to the WHO definition for physical distancing.

## DISCUSSION

Our study reports observed mask-wearing, physical-distancing, and handwashing behaviors of 4,736 individuals in indoor public spaces in eastern DRC. This is the first published study, to our knowledge, to observe all three of these behaviors together in indoor public spaces. This work builds on previous studies that have focused on self-reported preventive measures for COVID-19 transmission, and builds an evidence base on the prevalence of these preventive measures in a sub-Saharan African setting. Given the growing number of confirmed COVID-19 cases and deaths in the DRC, and the low COVID-19 vaccination rates (1.2%) in the country, COVID-19 preventive hygiene measures are essential to limit pandemic spread in this setting.^1^ However, we observed that despite government mandates and recommendations encouraging these preventive hygiene measures, fully covered mask-wearing (15%), physical-distancing (22%), and handwashing with a cleansing agent (10%) percentages in indoor public spaces were low. Furthermore, only a third of the handwashing stations in public indoor spaces had water and a cleansing agent present, and none of the handwashing stations indicating that they contained chlorine had levels that met CDC guidelines. In addition, few indoor public spaces had signs on mask wearing or COVID-19 at their entrances (17%). The fines and increased enforcement of the government mandate on mask wearing in public spaces in August 2021 resulted in significantly greater mask wearing practices. This result suggests that increased enforcement with fines could be a promising approach to increase fully covered mask wearing in indoor public spaces. The findings from this study demonstrate the urgent need for effective COVID-19 hygiene preventive response programs developed through formative research and community engagement to increase preventive hygiene measures against COVID-19.

There are a few previous studies from the DRC that have assessed COVID-19 preventive hygiene behaviors.^27^^–^^31^ Those studies found large variations in the self-reported rates of COVID-19 preventive hygiene behaviors.^27^^–^^31^ An in-person survey of health-care workers in the cities of Lubumbashi, Kamina, and Mbuji-Mayi found that 50% of respondents reported consistent mask use when leaving home.^30^ A web-based survey through Facebook, WhatsApp, and e-mail across the DRC found that 69% of respondents reported wearing a face mask when outside, 43% reported observing physical-distancing of 1.5 to 2-m, and 37% reported disinfecting hands immediately after coughing or sneezing, although the authors note these numbers are likely overestimates.^27^ Another online survey using Google forms of adults across the DRC older than 20 years found that 30% of those surveyed washed their hands after sneezing, and 2% followed mandatory mask-wearing.^29^ Two studies in the DRC conducted structured observations of COVID-19 preventive hygiene behaviors. The first, in Kinshasa, examined photographs from locations such as groceries, markets, and commodity food distribution centers^11^; the second took place in the public markets of three cities and one town in the former Katanga Province.^13^ Both occurred in April 2020. Mask wearing was less than 4% in these studies, much lower than self-reported behavior. These findings were lower than the 16% fully covered mask wearing in indoor public spaces found in our study. In the study conducted in Kinshasa, only half the markets had a handwashing station present,^11^ similar to our study.

The low rates of fully covered mask-wearing inside schools and universities (< 5%) and in health facility wards and religious establishments (< 30%) observed in our study is concerning, given the extended period of time individuals often spend in these locations. In our study, banks had the greatest percentage of indoor fully covered mask-wearing behavior. This was likely a result of the police who were often present at banks and were asked to enforce fully covered mask wearing. The low percentage of fully covered mask-wearing inside health facilities wards and main entrances is particularly concerning as well, given that many individuals are coming in for the treatment of illness, with some individuals that may be immunocompromised and therefore more susceptible to COVID-19 infection. The low mask-wearing percentage at this location is despite health facilities having the greatest percentage of COVID-19/mask-wearing signage. This finding suggests that further enforcement protocols need to be put in place by health facility administrators at these locations. More than 40% of mask-wearing behavior was partially covered mask wearing, where either the mouth, nose, or both were uncovered. This finding emphasizes the importance of promoting fully covered mask wearing, rather than “mask wearing” only.

In this study, we used both 5-hour structured observations at the entrances of indoor public spaces and rapid structured observations inside these spaces to observe mask-wearing and physical-distancing behaviors. Both measures provide complementary information. Five-hour structured observations at entrances provide valuable information on compliance with government mandates and recommendations by establishments on handwashing with a cleansing agent and fully covered mask wearing when individuals are entering these locations. Rapid structured observations, meanwhile, provide valuable information on mask-wearing and physical-distancing behaviors when individuals are inside these locations, and this technique has the major advantage of the data being quick to collect. Having both observation methods also allows for observing differences in mask-wearing behavior between these two spaces. For example, to determine whether, once inside, individuals take off their mask, as was the case for gyms and physical therapy offices. This was likely because individuals exert themselves during physical activity.

Overall, fully covered mask wearing was very similar at entrances and inside indoor public spaces, at 16% and 15%, with large differences only being observed at three locations: shops, gyms, and physical therapy offices. Given our finding on low mask-wearing behavior inside gyms and physical therapy offices, where patrons and patients are engaging in physical activity, we would recommend strong enforcement of physical-distancing in these settings, and for staff and providers to wear masks with greater protection (such as N95 or K95 masks, if available) fully covering the nose and mouth. In saunas, patrons likely took off their mask because of the high temperatures. In shops, we suspect the change from 7% in entrances to 32% inside was attributed to shop staff telling patrons to put on their mask upon entry. For overall physical-distancing, there were large differences between entrances (42%) and inside spaces (22%). This finding needs to be investigated further. If this percentage is a result of overcrowding, occupancy should be limited in these indoor public spaces and physically distanced waiting lines outside if capacity is reached. We recommend that evaluations conducted to assess mask wearing and physical-distancing behaviors use both rapid observations in indoor spaces and extended observations at entrances of locations to ensure these COVID-19 preventive hygiene behaviors are fully captured. Future studies should evaluate whether shorter durations for structured observations (e.g., 1 hour) can be used to replace 5-hour structured observations to save time and costs.

Previous studies in African settings have found barriers to high adherence of COVID-19 preventive hygiene behaviors, including lack of trust in the efficacy of these measures to reduce COVID-19, lack of trust that COVID-19 exists in Africa, and limited space to comply with physical-distancing requirements.^32^^–^^34^ These findings indicate the need for programs focusing on COVID-19 preventive hygiene behaviors to be tailored to the local context to ensure programs are targeting the contextual, psychosocial, and technological factors influencing adherence to the behavioral recommendations. Increased enforcement is also needed of government mandates on fully covered mask wearing, physical-distancing, presence of handwashing stations with water and a cleansing agent, and handwashing with a cleansing agent at indoor public spaces. This includes enforcing fines for lack of compliance to both establishments and individuals. Additional government mandates are likely also needed on maximum occupancy in public indoor spaces, which would allow for physical-distancing requirements to be met in otherwise crowded spaces, requiring that signs be present in indoor public spaces indicating that fully covered mask-wearing and handwashing with a cleansing agent are required upon entry. To prevent COVID-19 transmission in eastern DRC, a multilevel approach will likely be most effective—one that focuses on tailored COVID-19 preventive hygiene programs through community engagement at the community level and increased enforcement of government mandates at indoor public spaces.

There are no studies, to our knowledge, similar to our study that conducted structured observation of mask-wearing, physical-distancing, and handwashing together for communicable diseases such as avian or swine influenza, or other types of respiratory infections. Nor are there studies that investigated how assessing these measures together could inform safety compliance or the suppression of respiratory infection transmission. There are also no studies, to our knowledge, that evaluated whether fines lower COVID-19 transmission. These studies will be important for determining how to implement effective COVID-19 preventive hygiene programs.

This study has several strengths, the first of which is its observational design, which included both 5-hour structured observations at entrances of public indoor spaces and rapid observations inside these locations. Most published studies to date focus on self-reported COVID-19 preventive hygiene behaviors. Second is the focus on indoor public spaces, which have a greater COVID-19 transmission risk than outdoor spaces.^35^ However most observational studies in low- and middle-income countries have focused on outdoor locations.^11^^,^^16^^,^^17^^,^^23^ The third strength is the large sample size (> 4,700 individuals) and the 15 different types of indoor public spaces included. Most studies only included one or two location types.^7^^–^^9^^,^^11^^–^^16^^,^^18^^–^^23^ The fourth is including three types of COVID-19 preventive hygiene behaviors rather than focusing on only mask wearing, physical-distancing, or handwashing with a cleansing agent alone.

This study has some limitations. First, we did not record whether individuals entering and inside public indoor spaces were customers, patrons, staff, patients, or providers. In our attempt to be the least intrusive as possible, we did not capture this information. Second, we did not capture information on whether there was an individual standing at the entrance of indoor public spaces informing individuals that fully covered mask wearing and handwashing with a cleansing agent were required upon entry. This information would be valuable to collect in future studies. Third, we focused our study in an urban setting. Future studies should investigate these COVID-19 preventive hygiene behaviors in rural settings in the DRC and globally.

## CONCLUSION

This study presents rigorous methods using structured observations and spot checks that can be used for the evaluation of COVID-19 preventive hygiene programs in indoor public spaces in settings globally. Our work builds on previous studies that have focused primarily on self-reported COVID-19 preventive hygiene behaviors, and studies focusing on structured observations in outdoor public spaces only. We recommend that evaluations conducted to assess mask-wearing, physical-distancing, and handwashing behaviors use spot checks and structured observations at entrances and inside indoor public spaces to allow for a comprehensive assessment of these behaviors.

## Supplemental files


Supplemental materials
