# Supplementary material for: Methods to Evaluate COVID-19 Preventive Hygiene Programs: Observed Mask Wearing, Handwashing, and Physical Distancing Behaviors in Public Indoor Spaces in Democratic Republic of the Congo
Source: Am J Trop Med Hyg. 2022 Oct 3;107(5):1083–90. doi: 10.4269/ajtmh.22-0214 (PMC9709030; doi:10.4269/ajtmh.22-0214)
Supplement: Supplementary file 1 [file tpmd220214.SD1.pdf]

Supplemental Table 1. Presence of COVID-19/mask wearing signs and handwashing signs at spot check locations by location type

		**COVID-19/Mask wearing signage**		**Handwashing signage**	
**Location Type**	Total locations	n	%	n	%
Restaurant	10	0	0	1	10
Shop	10	0	0	0	0
Sauna	6	0	0	0	0
Physical therapy office	10	0	0	0	0
Supermarket	12	1	8	1	8
School	11	1	9	1	9
University	10	1	10	1	10
Gym	9	1	11	1	11
Religious establishment	14	2	14	2	14
Beauty salon	10	2	20	2	20
Office	11	3	27	3	27
Large retail center	10	3	30	3	30
Bank	10	3	30	2	20
Health facility main entrance	16	6	38	6	38
Health facility ward entrance	12	5	42	6	50
Overall	161	28	17	29	18

Percentages indicate the percent of locations with sign visible and legible at the location.
Religious establishments included 7 protestant or catholic churches and 1 mosque.
